# A patient‐derived explant (PDE) model of hormone‐dependent cancer

**DOI:** 10.1002/1878-0261.12354

**Published:** 2018-08-16

**Authors:** Margaret M. Centenera, Theresa E. Hickey, Shalini Jindal, Natalie K. Ryan, Preethi Ravindranathan, Hisham Mohammed, Jessica L. Robinson, Matthew J. Schiewer, Shihong Ma, Payal Kapur, Peter D. Sutherland, Clive E. Hoffmann, Claus G. Roehrborn, Leonard G. Gomella, Jason S. Carroll, Stephen N. Birrell, Karen E. Knudsen, Ganesh V. Raj, Lisa M. Butler, Wayne D. Tilley

**Affiliations:** ^1^ Freemasons Foundation Centre for Men's Health Adelaide Medical School University of Adelaide SA Australia; ^2^ South Australian Health and Medical Research Institute Adelaide SA Australia; ^3^ Dame Roma Mitchell Cancer Research Laboratories Adelaide Medical School University of Adelaide SA Australia; ^4^ Department of Urology UT Southwestern Medical Center at Dallas TX USA; ^5^ Knight Cancer Early Detection Advanced Research Center Oregon Health and Science University Portland OR USA; ^6^ Transcription Factor Laboratory Cancer Research UK Cambridge Institute Cambridge University UK; ^7^ Kimmel Cancer Center Thomas Jefferson University Philadelphia PA USA; ^8^ Urology Unit Surgical Specialties Service Royal Adelaide Hospital SA Australia; ^9^ Breast Clinic Burnside War Memorial Hospital Toorak Gardens SA Australia; ^10^ Department of Urology Thomas Jefferson University Philadelphia PA USA

**Keywords:** *ex vivo* culture, patient‐derived explant, preclinical tumor model

## Abstract

Breast and prostate cancer research to date has largely been predicated on the use of cell lines *in vitro* or *in vivo*. These limitations have led to the development of more clinically relevant models, such as organoids or murine xenografts that utilize patient‐derived material; however, issues related to low take rate, long duration of establishment, and the associated costs constrain use of these models. This study demonstrates that *ex vivo* culture of freshly resected breast and prostate tumor specimens obtained from surgery, termed patient‐derived explants (PDEs), provides a high‐throughput and cost‐effective model that retains the native tissue architecture, microenvironment, cell viability, and key oncogenic drivers. The PDE model provides a unique approach for direct evaluation of drug responses on an individual patient's tumor, which is amenable to analysis using contemporary genomic technologies. The ability to rapidly evaluate drug efficacy in patient‐derived material has high potential to facilitate implementation of personalized medicine approaches.

**Abbreviations**
ANOVA: analysis of variance
AR: androgen receptor
BrdU: 5‐bromo‐2‐deoxyuridine
ChIP: chromatin immunoprecipitation
E2: estradiol
ERα: estrogen receptor alpha
H&E: hematoxylin and eosin
LHRH: luteinizing hormone‐releasing hormone
PDE: patient‐derived explant
PDX: patient‐derived xenograft
PGR: progesterone receptor
PSA: prostate‐specific antigen
RARA: retinoic acid receptor‐α
RNAi: RNA interference
shRNA: short hairpin RNA

## 1 Introduction

Preclinical models that accurately replicate the native architecture and microenvironment of primary human tumors are urgently needed to improve our understanding of basic tumor biology and to facilitate the development of improved therapeutic approaches (Thompson *et al*., 2008). The use of immortalized human cell line models for investigating novel therapies *in vitro* or *in vivo* is convenient but also a major reason for the high failure of new drugs entering clinical trials. To move toward more clinically relevant model systems, researchers have adopted patient‐derived approaches such as organoids (Drost *et al*., 2016; Gao *et al*., 2014) and xenografts (PDX) (Lawrence *et al*., 2013; Wang *et al*., 2005; Whittle *et al*., 2015). We report an alternative approach of culturing freshly resected breast and prostate cancer tissue as patient‐derived explants (PDE). *Ex vivo* culture of human prostatic tissue has been used since the 1970s with varying degrees of success, and protocols ranging from total immersion of tissue pieces in medium to culture of tissue pieces or slices on grid or sponge scaffolds, reviewed by Centenera *et al*. (2013). Despite this long‐standing history and general acknowledgment of the potential of *ex vivo* cultured tissues to increase the clinical relevance of laboratory research (Centenera *et al*., 2013; Kim, 2005; Pretlow *et al*., 1995; Risbridger *et al*., 2018; Vescio *et al*., 1991), the PDE method has not been widely adopted to study solid tumors. The purpose of this study was to highlight advantages of the PDE model and demonstrate how it can be applied to interrogate hormone‐dependent cancers such as those of the breast and prostate.

## 2 Materials and methods

### 2.1 Ethical approval for research using human tissue

All research conducted in this study conformed to the standards set by the Declaration of Helsinki.

#### 2.1.1 Australia

The use of freshly resected human tissue samples for this study was approved by the Human Research Ethics Committees of the University of Adelaide, the Royal Adelaide Hospital, and Burnside War Memorial Hospital. All material is collected with written informed consent from patients, and data are de‐identified according to National Health and Medical Research Council of Australia guidelines for human research. Prostate tissues were collected as part of the Australian Prostate Cancer BioResource.

#### 2.1.2 United States

Prostate tissues from UT Southwestern Medical Center at Dallas and Thomas Jefferson University hospitals were obtained with written informed consent from patients undergoing radical prostatectomy for high‐volume cancer, under Institutional Review Board‐approved protocols for the respective institutions.

Clinical characteristics and histopathology of patients who donated tissue to this study are outlined in Table S1.

### 2.2 PDE culture of solid tumors

**Figure 1:**
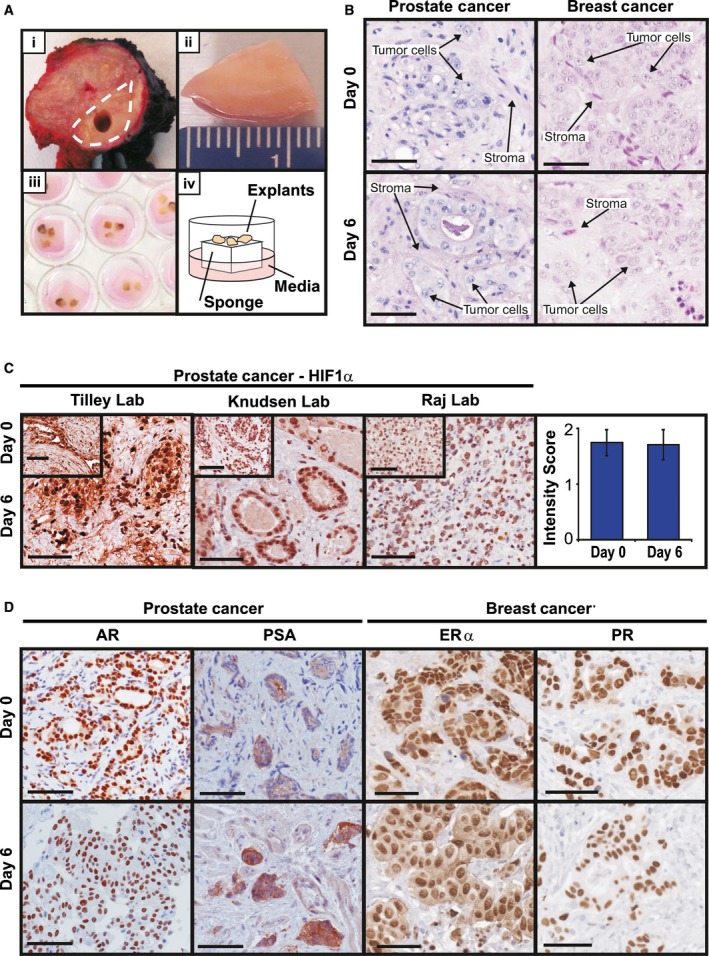
PDE culture sustains tissue morphology, viability, and endocrine signaling. (A) PDE tissue culture method. (i) Following surgery, a core of tumor tissue is removed by a pathologist (tumor area demarcated by broken white line), (ii) the tumor sample is dissected into 1‐mm^3^ fragments, (iii) cultured in 24‐well plates on a gelatin sponge sitting in media, allowing direct comparison of treatments in matched tumor tissue, and (iv) schematic diagram of PDE setup. (B) Representative hematoxylin and eosin staining of PDEs from primary prostate and breast tumors, showing maintenance of gross morphology following 6 days in culture. Arrows indicate examples of tumor cells and surrounding stroma. (C) HIF1α staining from three independent laboratories showed no significant difference in prostate cancer tissue oxygenation after 6 days of PDE culture. Staining intensity was manually assessed by a single pathologist (P. Kapur). Data are presented as mean ± SEM,* n* = 3. (D) Expression and signaling of steroid receptors critical for prostate and breast carcinogenesis were maintained in PDEs cultured in complete media for 6 days, as demonstrated by immunostaining for AR and the AR‐regulated protein PSA in prostate PDEs, and ERα and the ERα‐regulated protein PGR in breast cancer PDEs. Scale bars represent 50 μm.

The PDE technique utilized in this study employs a gelatin sponge platform (Centenera *et al*., 2013) (Fig. 1A). This method was selected for two key reasons: firstly, the use of a substrate for explant culture prevents cellular outgrowth that is frequently observed when tissues are cultured without support and completely submerged in media (Pretlow *et al*., 1995; Varani *et al*., 1999); secondly, the gelatin sponge used in this study is a commercial medical device developed for hemostasis (Ferrosan, 2014) that is readily available, cost‐effective, and simple to use, making the method feasible for widespread adoption in translational cancer research laboratories.

Figure 1A illustrates the PDE method using prostate tumors as an example. Following surgical tissue removal, the surgeon or clinical pathologist resects a sample of the presumed malignant or nonmalignant region and the specimen is transported to the research laboratory in cold phosphate‐buffered saline on ice, typically within 1–2 h of surgery. Under sterile conditions, tissue was placed onto the lid of a 10‐cm plate along with the saline it was transported in. Using a surgical blade, a 1‐mm‐thick longitudinal section of the tissue sample is cut and placed into neutral‐buffered formalin for paraffin embedding. This tissue is called the Day 0 sample and is used to determine the cancer content of the tissue following staining with hematoxylin and eosin (H&E). The remaining tissue is dissected into 1‐mm^3^ pieces, called explants, and placed in triplicate or quadruplicate (depending on amount of tissue received) on top of a media‐soaked gelatin sponge (Ethicon; Johnson & Johnson, Somerville, NJ, USA) inside the wells of a 24‐well plate containing 500 μL RPMI 1640 medium containing 10% fetal bovine serum, 1× antimycotic/antibiotic solution, 0.01 mg·mL^−1^ hydrocortisone, and 0.01 mg·mL^−1^ insulin. The appropriate vehicle, treatment, or shRNA was added directly to the media inside the appropriate tissue culture well at the indicated concentrations, allowing direct comparison of treatments and controls. Explants were cultured at 37 °C and 5% CO_2_ for various time points and then formalin‐fixed and paraffin‐embedded, snap‐frozen, or preserved in RNAlater (Invitrogen, San Diego, CA, USA) depending on the desired downstream analysis. For assessment of BrdU incorporation, BrdU (10 μm) was added to the culture medium 2 h (prostate) or 24 h (breast) prior to harvest.

In our collective experience, all tissues containing epithelial cells can successfully be cultured, and the limiting factor for analysis is instead the presence of sufficient numbers of epithelial (for benign tissue studies) or malignant cells (for cancer tissue studies). For this reason, routine hematoxylin and eosin (H&E) staining of all Day 0 and cultured tissues is an essential part of our protocol to confirm the presence and approximate percentage of benign/malignant cells within the specimens before proceeding with further analyses. Between the three prostate cancer laboratories, our collective experience indicates that approximately 10% of tissues from high‐volume disease and 20–30% of tissues from low‐volume disease do not contain malignant cells, and this is largely due to sampling.

### 2.3 Immunohistochemistry

Immunohistochemical staining was performed on 2‐ to 4‐μm sections that were deparaffinized, rehydrated, and blocked for endogenous peroxidase before being subjected to heat‐induced epitope retrieval. Breast tissues were additionally treated to block for endogenous biotin. Sections were blocked and incubated with the appropriate primary and secondary antibodies, then developed using 3‐3′‐diaminobenzidine chromogen, and counterstained with hematoxylin. Appropriate positive and negative controls were included in all runs. Table S2 lists pertinent information for all antigens. Immunostaining in the Tilley laboratory was performed manually, and in the Raj laboratory, immunostaining was performed on the DAKO autostainer (DakoCytomation, Carpinteria, CA, USA). Tissues cultured by the Knudsen laboratory were stained by the Raj laboratory. The percent positivity and intensity of nuclear staining for each antigen were quantified manually by an independent pathologist or observer, who was blinded to the treatments/conditions.

### 2.4 RNA extraction

Tissues preserved in RNAlater (Qiagen, Hilden, Germany) were homogenized in ice‐cold PBS with a Precellys 24 Tissue Homogenizer (Bertin Technologies, Montigny‐le‐Bretonneux, France), and RNA was extracted using Trizol according to the manufacturer's instructions. DNAse treatment was performed using the TurboDNase kit (Ambion, Austin, TX, USA) according to manufacturer's instructions. RNA was quantified using a Nanodrop 1000 spectrophotometer (Thermo Fisher Scientific, Waltham, MA, USA). Reverse transcription was performed on 400 ng total RNA using the iScript kit (Bio‐Rad Laboratories, Hercules, CA, USA) according to manufacturer's instructions.

### 2.5 Quantitative real‐time PCR

qPCR was performed using 2 μL of cDNA from whole tissue extracts and SYBR green for prostate explants, and gene‐specific TaqMan assays were performed for breast explants. Primers and assay IDs are listed in Table S3. Relative gene expression was calculated using the ΔCt method. Expression of *PSA* in prostate explants was normalized to *PPIA*,* L19*,* TUBA1B*,* ALAS1*, and *GAPDH*. Progesterone receptor (PGR) expression in breast explants was normalized to IPO8 and PUM1.

### 2.6 Ablation of AR by shRNA

The shAR and shControl lentiviral constructs were packaged using Lenti‐X HT (Open Biosystems, Dharmacon Inc., Lafayette, CO, USA) according to the manufacturer's instructions and added to the tissue culture medium for 48 h. Lentiviral media was then removed and replaced with fresh media containing no lentivirus and cultured for an additional 48 h. Tissues were harvested and formalin‐fixed prior to immunohistochemistry staining for androgen receptor (AR) and prostate‐specific antigen (PSA) as described above.

### 2.7 Chromatin immunoprecipitation followed by massively parallel DNA sequencing (ChIP‐seq)

Breast cancer PDEs cultured in complete medium containing vehicle (EtOH; 0.1%), 17β‐estradiol (E2; 10 nm), or synthetic PGR agonist R5020 (10 nm) for 72 h were harvested, crosslinked, and processed for ChIP‐seq analysis as described previously (Ross‐Innes *et al*., 2012). The estrogen receptor alpha (ERα) HC‐20 antibody (Santa Cruz Biotechnologies, Dallas, TX, USA) was used for immunoprecipitation of ERα from PDE tissue lysates. Sequences generated by an Illumina HiSeq 2000 were processed by the Illumina analysis pipeline version 1.6.1 and aligned to the Human Reference Genome (hg19) using BWA version 0.5.5 (Li and Durbin, 2009). Reads were filtered by removing those with a BWA alignment quality score less than 15. Enriched ERα binding regions were identified by comparing ERα ChIP DNA samples to total ChIP input DNA. ER ChIP‐sequencing was then performed from each PDE tissue and treatment. Peak calling was performed using MACS2 version 1.4.0rc2 (Zhang *et al*., 2008).

### 2.8 Statistical analysis

Data are displayed as the mean ± standard error. Differences were determined using Student's *t*‐test or one‐way analysis of variance (ANOVA). The correlation between Ki67 and PSA expression was analyzed using the Spearman correlation test with an accompanying *P* value. All statistics and generation of heat map were performed using graphpad prism Software version 7.02 (GraphPad Software, La Jolla, CA, USA). A *P*‐value ≤ 0.05 was considered statistically significant.

## 3 Results

### 3.1 Morphology, viability, and molecular signaling are sustained in PDEs

Histopathological evaluation of tissue architecture and cellular appearance demonstrated that the morphology of PDEs cultured for up to 6 days on gelatin sponges is consistent with the original, uncultured (i.e., Day 0) tumor tissue, as illustrated by representative H&E staining of prostate cancer and breast cancer (Fig. 1B). Tumor cells are present in the surrounding stroma, demonstrating maintenance of the tumor microenvironment (Fig. 1B). Immunohistochemical staining for the hypoxia‐inducible factor (HIF)‐1α showed no evidence of hypoxia in PDEs cultured using the sponge method (Fig. 1C). Importantly, equivalent HIF‐1α staining was observed in prostate tumors cultured in three independent laboratories (Fig. 1C) and evaluation of serial sections of individual tissues indicated no discernible difference in histology or antigen staining between the air or sponge interface (data not shown).

The AR and ERα are critical drivers of prostate and breast cancer, respectively; therefore, AR and ERα were assessed as examples of major oncogenic signaling pathways in the PDE model. Epithelial cell positivity for immunoreactive AR and ERα was sustained within malignant prostate and breast PDEs, respectively, for up to 6 days in complete media (Fig. 1D). Expression of the androgen‐regulated protein, PSA, and the estrogen‐regulated protein PGR, indicative of functional signaling by AR and ERα, respectively, was also evident after 6 days of culture (Fig. 1D).

### 3.2 Proliferative capacity of PDE tumor tissue

**Figure 2:**
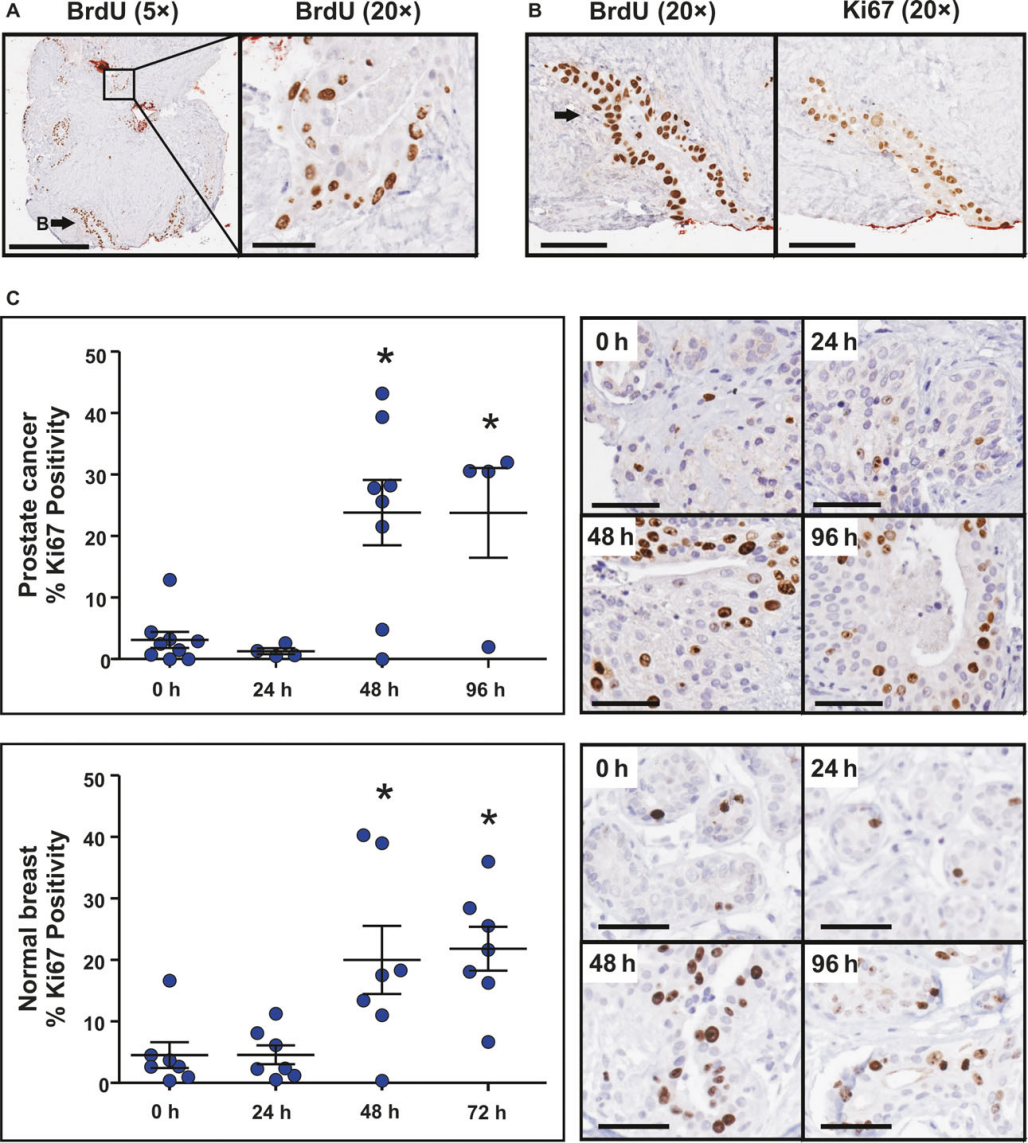
Proliferative capacity of PDEs. (A) *De novo* proliferation of tumor cells in PDE cultured tissues is demonstrated by BrdU uptake in a representative prostate cancer explant. (B) The distribution of BrdU uptake is similar to expression of the proliferative marker Ki67 as shown in a representative prostate cancer PDE. (C) Representative images and quantitation of Ki67 immunostaining in prostate (*n* = 9) and breast (*n* = 8) tissue at Day 0 and in PDEs cultured for up to 96 h in complete media. *ANOVA: Day 0 *versus* time points, *P* = 0.0007 for prostate; *P* = 0.0013 for breast. All scale bars represent 50 μm.

Cellular uptake of the thymidine analog 5‐bromo‐2‐deoxyuridine (BrdU; Fig. 2A) and its nucleoside analog 5‐ethynyl‐2′‐deoxyuridine (Fig. S1) from the culture media was observed in a subset of prostate tumors, indicating that cultured tissues have the capacity for *de novo* proliferation. BrdU uptake was evident throughout the PDE samples (Fig. 2A) and was concordant with Ki67 positivity (Fig. 2B). Ki67 positivity in breast and prostate explant tissues was investigated further to assess suitability of the PDE model to evaluate proliferative responses to hormonal stimuli, growth factors, or therapeutic agents. The number of Ki67‐positive epithelial cell nuclei (i.e., the cellular proliferative index) in Day 0 tissues ranged from 0 to 16% and increased to a range of 0 to 43% (*P* < 0.05) in matched tissues after 48 h of culture and remained stable for at least 96 h (Fig. 2C). The time‐dependent increase in proliferation observed here and in previous organotypic culture models (Nevalainen *et al*., 1993; Olbina *et al*., 1998) is likely due to growth‐promoting factors in the media or to the release of an inhibitory serum influence that was present *in vivo*, but absent *ex vivo*.

### 3.3 Modulation of AR signaling in prostate cancer PDEs

**Figure 3:**
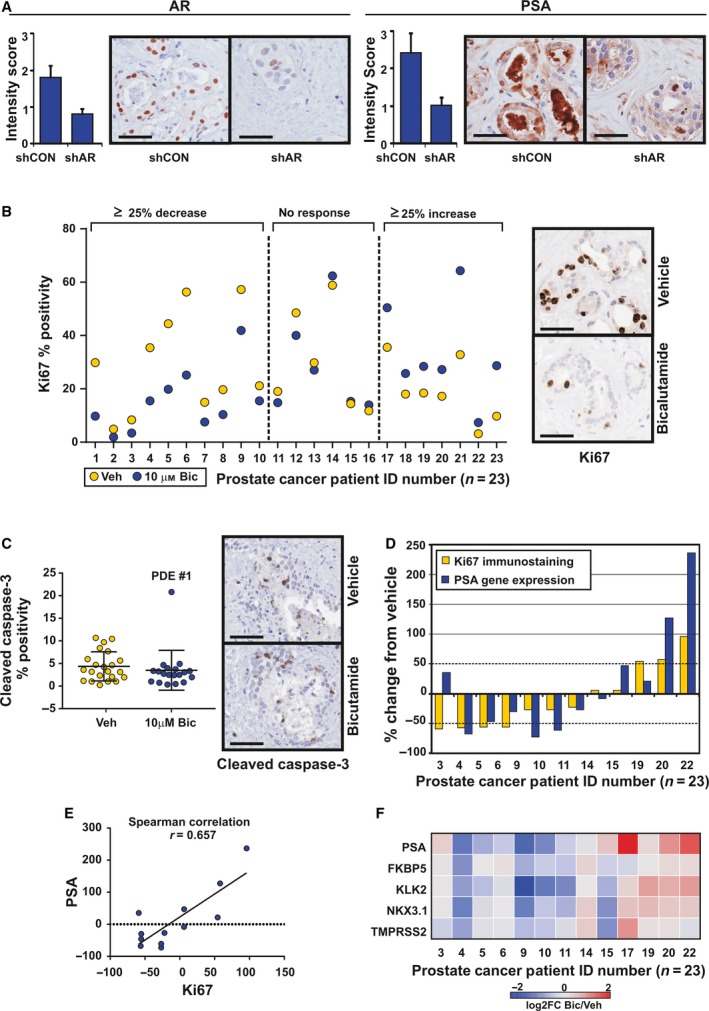
Modulation of AR signaling in prostate cancer PDEs. (A) Steady‐state protein levels of AR and the AR‐regulated protein PSA were knocked down in prostate cancer PDEs (*n* = 3) cultured in media containing lentiviral‐based shRNA directed against AR (shAR) compared with scrambled control (shCON). Scale bars represent 50 μm. (B) Quantitation and representative images of Ki67 immunostaining in PDEs derived from 23 patients following 48 h culture with vehicle control or bicalutamide (10 μm). A response to bicalutamide was considered significant when treatment induced a change from vehicle of ≥ 25%. Scale bars represent 50 μm. (C) Quantitation and representative images of cleaved caspase‐3 immunostaining in PDEs derived from 23 patients following 48‐h culture with vehicle control or bicalutamide (10 μm). Data are presented as mean ± SEM. Scale bars represent 50 μm. (D) Water fall plot of percent change in *PSA* gene expression and Ki67 immunostaining from a subset of PDEs from (A) treated with vehicle control or bicalutamide (*n* = 12). (E) Scatterplot of the data from D showing a positive correlation between Ki67 and PSA with Spearman's *r* = 0.657 (*P* < 0.05). (F) Heat map visualization of qRT‐PCR analysis of classic AR‐regulated transcripts in bicalutamide‐treated PDEs compared to vehicle (*n* = 12).

AR is the driving transcription factor in prostate cancer and the primary therapeutic target for systemic treatment. To demonstrate modulation of this critical signaling pathway, a lentiviral‐based shRNA approach was used for isogenic suppression of *AR* gene expression. Lentiviral transduction of cultured prostate PDEs with an *AR*‐directed shRNA for 48 h decreased the steady‐state protein levels of AR by approximately 50% (Fig. 3A). Importantly, AR inhibition resulted in a 40% decrease in expression of the AR‐regulated protein PSA (Fig. 3A), which demonstrates that the AR signaling pathway is functional in PDE tissues.

Clinical inhibition of the AR signaling pathway is achieved through androgen deprivation therapy or AR antagonists (Attard *et al*., 2011). To demonstrate capacity of the PDE model for evaluating therapeutics, prostate cancer PDEs were treated with the clinically used AR antagonist bicalutamide. Proliferative response was assessed using the immunohistochemistry marker Ki67, with a response considered significant when treatment induced a change from vehicle of ≥ 25%. Proliferative responses were observed after only 48 h of culture; therefore, this time point was selected to allow short‐term, high‐throughput preclinical testing to be performed. Similar to Fig. 2C, a large range of baseline Ki67 (2‐58%) was observed across the cohort (Fig. 3B). In prostate cancer PDEs cultured in the presence of 10 μm bicalutamide for 48 h, the proliferative index was reduced in 10/23 tissues (44%), increased in 6/23 tissues (26%), and had no significant effect on proliferation in 7/23 tissues (30%) compared to vehicle‐treated PDEs (Fig. 3B). Similar heterogeneity in response was observed with the newer generation antagonist enzalutamide in a smaller, independent cohort of PDEs (Fig. S2). A change in apoptosis was not observed in PDEs cultured for 48 h with 10 μm bicalutamide (Fig. 3C). Collectively, these findings illustrate the heterogeneity of clinical samples and demonstrate the importance of using a model that reflects this natural variation when evaluating solid tumors. A critical aspect of this research will be relating the observed heterogeneity with clinical outcomes of these patients, but we were unable to assess this in the current study. To date, only one patient (Fig. 3A patient no. 18) from our cohort of predominantly low‐to‐intermediate risk patients (median follow‐up time of 48.3 months; range 23–64.4 months) has experienced biochemical recurrence (BCR). This is consistent with the reported 5‐year BCR rate of 10–20% in low‐to‐intermediate risk patients undergoing robotic‐radical prostatectomy (Diaz *et al*., 2015) and highlights the need for longer‐term follow‐up to correlate PDE results with individual patient outcomes. A more feasible clinical context to ascertain whether PDE culture is correlated with clinical outcome is neoadjuvant clinical trials that allow direct comparison of pre‐ and post‐treated tumors.

In a subset of 12 PDEs from Fig. 3B, where sufficient matched tissue was available to extract RNA, changes in *PSA* gene expression in response to bicalutamide treatment were evaluated by qPCR. Similar to Ki67, heterogeneity in *PSA* response to bicalutamide was observed across the 12 explants (Fig. 3D). Importantly, in 10/12 tissues Ki67 positivity and *PSA* expression increased or decreased concordantly, demonstrating a significant positive association (*r* = 0.657; *P* = 0.0238; Fig. 3E). Transcript analysis of additional prototypical AR‐regulated genes *FKBP5*,* KLK2*,* NKX3.1,* and *TMPRSS*2 shows a similar pattern of expression to PSA and Ki67 (Fig. 3E), indicating general inhibition of androgen signaling by bicalutamide in prostate cancer PDEs. No correlation was observed between proliferative response and tumor grade, stage, or presurgery serum PSA. However, we acknowledge that a larger range of samples may be required to determine this conclusively as the majority of our specimens were of Gleason grade 7, stage PT3A/B, and PSA < 10 (Table S1).

### 3.4 Analysis of ERα signaling in breast cancer explants

**Figure 4:**
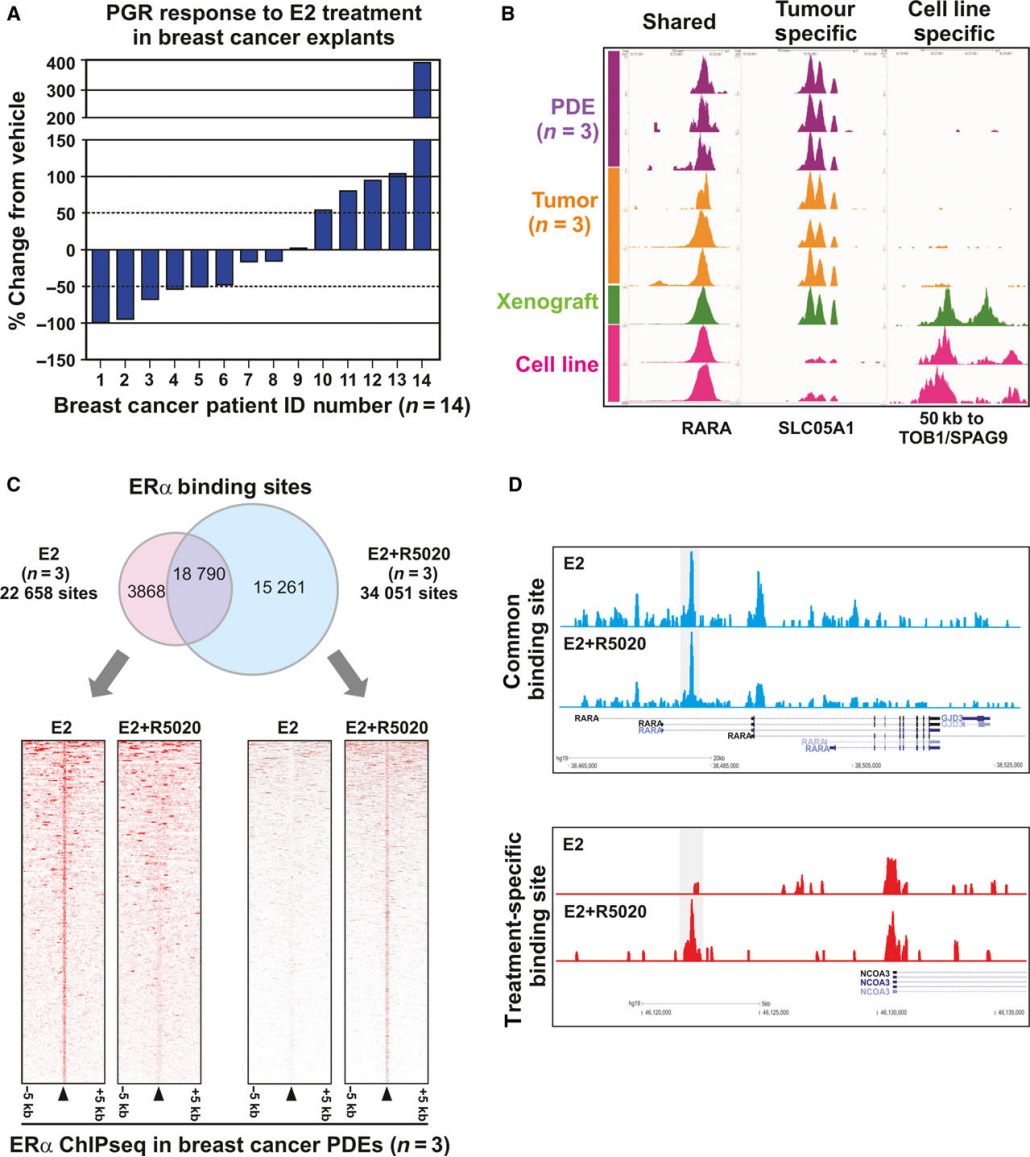
Modulation of ERα signaling in breast cancer PDEs. (A) qPCR analysis of PGR expression shows differential response to 10 nm E2 in breast cancer PDEs (*n* = 14). Samples with ≥ 50% change compared to vehicle were considered responsive. Data are presented as the mean ± SEM. (B) ChIP‐sequencing analysis of ERα binding sites in breast cancer PDEs (*n* = 3), untreated primary breast cancers (*n* = 3), an *in vivo* xenograft tumor grown from the ERα‐positive MCF7 cell line, and MCF7 cells cultured *in vitro* (*n* = 2). Shown are examples of ERα binding events that are shared by all models (RARA), present only in *in vivo* models (SLCO5A1) or present only in cell line models (TOB1/SPAG9). (C) Venn diagram showing the overlap of ERα binding sites identified in PDEs treated with E2 or E2+ R5020. Only ChIP‐seq peaks identified in at least two tumors were considered included. Heat map of treatment‐specific binding events from the Venn diagram. Data were centered at the top of the peak and visualized with a 5‐kb window around the peak. (D) ERα ChIP‐seq binding sites identified in E2− or E2+ R5020‐treated breast cancer PDEs. Examples of common binding sites (upper panel) and treatment‐specific binding (lower panel) sites are shown.

Estradiol (E2)‐activated ERα binds to *cis*‐regulatory elements of target genes such as the *PGR* (Ross‐Innes *et al*., 2012). Candidate gene analyses performed on RNA extracted from ERα‐positive PDEs (*n* = 14) cultured for 24 h with E2 revealed a range of responses to hormone treatment. E2 stimulation decreased *PGR* expression by ≥ 50% in 6/14 tissues (43%), increased *PGR* expression by ≥ 50% in 5/14 tissues (36%), and had no significant effect on *PGR* in 3/14 tissues (21%) compared to the matched vehicle controls (Fig. 4A). To further investigate ERα signaling, we used ChIP‐seq to evaluate E2‐treated breast cancer PDEs and compared whole‐genome ERα binding events with primary breast cancer tissues and traditional breast cancer models, including the most commonly used ERα‐positive breast cancer cell line, MCF7, grown *in vitro* or *in vivo* as xenografts. Figure 4B depicts an example ERα binding site shared by all models [retinoic acid receptor‐α (*RARA*)], a binding site identified in patient‐derived tissues only (*SLCO5A1*), and binding sites identified in cell line models only (50 kb to TOB1/SPAG9). These findings clearly highlight how cell line models do not accurately represent clinical breast cancer and the importance of utilizing tissue‐based models for interrogation of mechanisms of carcinogenesis.

We have previously shown that *PGR* is not only an ERα‐target gene but is also an ERα‐associated protein that can reprogram ERα DNA binding and transcriptional responses in breast cancer and, importantly, used the PDE model to study the transcriptome and growth effects of this ERα reprogramming by PGR (Mohammed *et al*., 2015; Singhal *et al*., 2016). To further investigate these findings, herein we report successful ERα ChIP‐seq in ERα‐positive breast cancer PDEs cultured with E2 in the presence or absence of synthetic PGR agonist R5020. The number of ERα binding events in each E2‐treated and E2 + R5020‐treated PDE is shown in Table S4. ERα binding could be mapped in all PDEs, but total peak intensity and the number of identified binding events differed between PDEs and treatments. To systematically quantify these observations, consensus ERα chromatin binding sites were compiled for each treatment by including peaks found in at least two of the three cases. Under estrogenic conditions alone, there were a total of 22 658 consensus ERα binding sites, and under estrogenic conditions with R5020 supplementation, there were a total of 34 051 consensus sites, with 18 790 shared sites between the two conditions (Fig. 4C). Further 3868 and 15 261 ERα binding sites were identified to be specific to either estrogen alone or estrogen plus R5020 treatment, respectively (Fig. 4C). Representative images of ERα chromatin binding at the RARA‐positive control locus demonstrate robust peaks common to both treatment conditions (Fig. 4D, upper panel), as well as binding peaks specific to individual hormone treatments, such as those observed 10 kb upstream of the nuclear receptor coactivator 3 (NCOA3) locus (Fig. 4D, lower panel).

## 4 Discussion

Patient‐derived explant tissue culture on a gelatin scaffold retains many features of human solid tumors, including the native microenvironment and cellular interactions that are critical for carcinogenesis but are lacking in many preclinical models. This study demonstrates that PDEs are not only viable in culture but can be manipulated using hormones, siRNA, or cancer drugs and that the response to those interventions can be assessed using an array of techniques, including immunohistochemistry, real‐time qRT‐PCR, and genomewide molecular analyses of cistromes. The PDE method is robust, as shown through observations of sustained tissue morphology, viability, and maintenance of critical cancer‐related signaling pathways in independent laboratories. It is also applicable to multiple solid tumor types; to date, the methodology has successfully been applied to all cancers tested, including some not represented in the current study (ovary, endometrium, renal, sarcoma; personal communication). The *de novo* cellular proliferation observed in cultured tissues indicates that the system is not static and makes this a particularly useful model to assess the growth inhibitory activity of new or emerging therapeutic agents, as demonstrated by our respective teams using PDEs from prostate cancer and breast cancer (Centenera *et al*., 2012; Comstock *et al*., 2013; Dean *et al*., 2012; de Leeuw *et al*., 2015; Schiewer *et al*., 2012).

One of the most exciting aspects of the PDE model is the potential to uncover information about the diversity of tumor biology that is not possible using cell lines or cell line xenografts due to their clonality. Observed changes in *PSA* and *PGR* expression in prostate and breast tumors as markers of AR and ERα signaling, respectively, reflected the natural tumor heterogeneity observed clinically for each tumor type examined (Arnedos *et al*., 2013). Variation in PSA response to bicalutamide, the most common antagonist used in locally advanced disease (Heidenreich *et al*., 2014), in prostate PDEs is consistent with outcomes of the TERRAIN and STRIVE clinical trials. As an AR antagonist, bicalutamide inhibits expression of AR‐regulated genes such as *PSA*, as well as genes involved in cellular proliferation, differentiation, and survival (Furr, 1996; Maucher and von Angerer, 1993). In TERRAIN and STRIVE, a serum PSA decline of ≥ 50% from baseline was achieved in 31% and 21% of bicalutamide‐treated patients, respectively (Penson *et al*., 2016; Shore *et al*., 2016). In our cohort of PDEs treated with bicalutamide, a similar rate of PSA decline was observed, with 25% of PDEs showing ≥ 50% decrease in *PSA* gene expression compared with vehicle treatment. *PSA* increase from baseline was also observed in a subset of patients in both clinical trials, which was again similar to our results where PSA increased ≥ 50% compared with vehicle treatment in 16% of cases.

Ki67 expression is a measure of cells in the active phase of the cell cycle and is a widely used marker of proliferating cells (Scholzen and Gerdes, 2000). Ki67 proliferative index is an independent predictor of prostate cancer outcomes (Fisher *et al*., 2013; Pollack *et al*., 2004; Zellweger *et al*., 2009); however, the wide range of Ki67 expression naturally observed in clinical prostate tumors and a lack of consensus on appropriate cutoff points have prevented utilization of Ki67 as a marker in the clinic (Penault‐Llorca and Radosevic‐Robin, 2017). Similarly, we observed wide variation in baseline Ki67 expression in PDE tissues, reflecting the diverse genetic heterogeneity of prostate tumors (Barbieri *et al*., 2013). Further, we observed a positive correlation between Ki67 immunostaining and androgen signaling in bicalutamide‐treated PDEs, consistent with the effect of bicalutamide on prostate tumor PDE proliferation being AR‐mediated. Despite observing significant effects of bicalutamide on PDE proliferative index, apoptotic response to bicalutamide was also evaluated, using the immunohistochemistry marker cleaved caspase‐3. We have previously reported apoptosis induction in prostate cancer PDEs cultured for 48 h with other therapeutic agents (Centenera *et al*., 2012). It is therefore likely that higher doses of bicalutamide than the 10 μm used in this study are required to induce apoptosis in prostate cancer PDEs.

Breast cancer is highly heterogeneous, clustering into 10 different molecular subgroups based on an integrated analysis of genomic aberrations and transcriptional profiling (Curtis *et al*., 2012). Tumors that express ERα represent the majority (≥ 70%) of all cases (Curtis *et al*., 2012). Assessment of ERα and PGR status by immunohistochemistry guides treatment decisions for breast cancer, as PGR is an ERα‐regulated gene and used as a biomarker of ERα activation (Lee and Gorski, 1996). We found that ERα signaling is not only sustained in breast cancer PDEs, but that the estrogenic response of ERα‐positive PDEs significantly varies in terms of *PGR* regulation. Although an established ERα‐regulated gene, *PGR* expression at the mRNA level can be influenced by factors that modulate the transcriptional activity of ERα (e.g., E2 metabolism, expression of transcriptional cofactors, degree of receptor phosphorylation) or regulate PGR expression independent of ERα (e.g., environmental levels of progesterone, insulin‐like growth factors), reviewed in Ref. (Grimm *et al*., 2016). Exposure to exogenous hormones through endocrine therapy or menopausal hormone therapy is another factor that affects ERα and PGR status (Ali and Coombes, 2002). Therefore, the heterogeneity we observed is likely representative of individual tissue microenvironments, and the PDE model provides an avenue to more accurately dissect how the tumor setting influences ERα signaling. In support of this concept, we used genomewide profiling of hormone‐treated breast cancer PDEs to capture ERα binding events and demonstrated reprogramming of ERα binding by synthetic progestin R5020, a phenomenon we recently reported using cell line models and clinical samples (Mohammed *et al*., 2015; Singhal *et al*., 2016).

Collectively, our data demonstrate the major advantage of the PDE model, which is the capacity for quantitative evaluation and comparison of different pharmacological agents in matched patient material. Traditionally, within‐patient comparisons have only been possible between pre‐ and post‐treatment samples from neoadjuvant studies or through utilization of diagnostic needle biopsies (Beltran *et al*., 2017). Obtaining this type of material is notoriously difficult without direct access to clinical trials or due to the limited amount of quality tissue available from biopsy after diagnostics are complete. The utilization of clinical material for profiling the genome, transcriptome, and proteome has remarkably advanced our understanding of the molecular features of breast and prostate cancer. The potential now offered by the PDE model is ‐omic analysis of matched treated and untreated samples using the PDE model. Our teams have published transcriptomic and proteomic analysis of PDE tissues (Armstrong *et al*., 2018; Nguyen *et al*., 2018; Pishas *et al*., 2014), and here, we demonstrate the added capacity for cistromics. Expanding the repertoire to include the lipidome, metabolome, kinome, and secretome will provide further critical insight into tumor biology and better define the mechanism of action of pharmaceutical agents preclinically, which will lead to the identification of more clinically relevant therapeutic targets and improved translation of research findings.

Preclinical models that more accurately represent human disease are needed to improve translational cancer research outcomes. The appreciation for patient‐derived models in this context is increasing rapidly in parallel with organoid and PDX techniques. The major difference between explant and organoid culture is that the latter are generated from tumors that are minced and enzymatically digested to dissociate cells for seeding in Matrigel (Gao *et al*., 2014), whereas explant tissue is cultured in its native format without disruption to the tissue architecture or tumor microenvironment. In this way, explants are more analogous to PDX models but without the significant associated costs, timeframes, and complication of infiltrating mouse stroma (Whittle *et al*., 2015). Another disadvantage of PDX models is that the take rate is biased toward more aggressive tumors such that engraftment may even be indicative of poor patient outcome (DeRose *et al*., 2013). The poor take rate of primary tumors means that PDX lines do not necessarily represent all disease states. In contrast, tissues from benign, primary, and advanced disease stages have been successfully cultured using the PDE technique with an extremely high take rate that is dependent on the presence of epithelial cells rather than the aggressiveness of the tumor cells. The PDE method therefore enables rapid, high‐throughput, and cost‐effective analyses of diverse human tumors and disease stages. We propose that incorporation of the PDE model into preclinical drug development programs will facilitate better selection of agents for clinical trials and provide biological insight into key molecular pathways of oncogenesis. The major criterion for implementation of the PDE model is ready access to fresh tissue samples, necessitating a strong collaboration between surgeons, pathologists, and scientists. The reliance on fresh tissue also means that PDEs cannot be passaged or revived from frozen material. What remains to be proven over the longer term is whether this approach can indeed predict *in vivo* clinical responses and lead to new, more effective cancer treatments or biomarkers of treatment response. Toward this goal, a recent study in non‐small‐cell lung cancer reported a significant correlation between cisplatin sensitivity in *ex vivo* cultured tissue and patient survival (Karekla *et al*., 2017). Future neoadjuvant clinical studies comparing pre‐ and post‐treatment tissues to include parallel *ex vivo* cultures of the pretreatment tumor tissues will validate the PDE model using clinically relevant end points. This will provide an exciting opportunity to investigate novel mechanisms of treatment resistance and identify biomarkers of treatment response that are essential for the realization of personalized cancer medicine.

## 5 Conclusion

PDE culture of breast and prostate tumors is a high‐throughput and cost‐effective technique that retains the tissue's native architecture, microenvironment, and key oncogenic drivers. This approach allows direct within‐patient comparison to rapidly evaluate efficacy of therapeutic agents in a personalized manner and is amenable to analysis using contemporary molecular technologies.

## Author contributions

MMC, SNB, LMB, and WDT developed the culture methodology. MMC, TEH, KEK, GVR, LMB, and WDT designed the study and prepared the manuscript. PDS, SNB, CEH, CGR, and LGG assisted with patient recruitment and clinical information and provided clinical samples. MMC, TEH, NKR, PR, JLK, MJS, CESC, and SM designed and performed experiments. MMC, TEH, HM, JLK, JSC, KEK, GVR, LMB, and WDT were involved in data analysis and interpretation. SJ and KP provided pathological expertise and analysis. All authors discussed results and provided comments on the manuscript.

## Supporting information


**Table S1.** Clinical and pathological characteristics of tumors used in this study.
**Table S2.** Summary of antibodies and conditions for immunohistochemistry.
**Table S3.** Primer sequences/Taqman Probe Assay IDs.
**Table S4.** Number of ER binding peaks identified by Mac Peak caller.
**Fig. S1.** Representative images of EdU staining in prostate cancer PDEs.
**Fig. S2.** Quantitation of EdU staining in prostate cancer PDEs.
