# Supplementary material for: A patient‐derived explant (PDE) model of hormone‐dependent cancer
Source: Mol Oncol. 2018 Aug 16;12(9):1608–22. doi: 10.1002/1878-0261.12354 (PMC6120230; doi:10.1002/1878-0261.12354)
Supplement: Supplementary file 1 — Table S1. Clinical and pathological characteristics of tumors used in this study. Table S2. Summary of antibodies and conditions for immunohistochemistry. Table S3. Primer sequences/Taqman Probe Assay IDs. Table S4. Number of ER binding peaks identified by Mac Peak caller. Fig. S1. Representative images of EdU staining in prostate cancer PDEs. Fig. S2. Quantitation of EdU staining in prostate cancer PDEs. [file MOL2-12-1608-s001.pdf]

Supplementary Table 1. Clinical and pathological characteristics of tumors used in this study.

Laboratory	Gender	Age	Histological type	Site	Gleason Grade	PSA at diagnosis	Pathological stage
Tilley	M	66	Adenocarcinoma	Prostate	4+3+5	6.1	PT3B
Tilley	M	69	Adenocarcinoma	Prostate	3+3=6	8.1	PT3A
Tilley	M	49	Adenocarcinoma	Prostate	3+4=7	10.0	PT3A
Tilley	M	72	Adenocarcinoma	Prostate	4+3	13.5	PT3A
Tilley	M	50	Adenocarcinoma	Prostate	3+4=7	6.0	PT2C
Tilley	M	61	Adenocarcinoma	Prostate	3+4=7	15.0	PT2C
Tilley	M	69	Adenocarcinoma	Prostate	3+4=7	12.0	PT2C
Tilley	M	66	Adenocarcinoma	Prostate	3+4=7	10.0	PT2C
Tilley	M	72	Adenocarcinoma	Prostate	3+3=6	10.3	PT3A
Tilley	M	53	Adenocarcinoma	Prostate	3+3=6	9.8	PT2C
Tilley	M	63	Adenocarcinoma	Prostate	3+4=7	14.0	PT3A
Tilley	M	50	Adenocarcinoma	Prostate	3+4=7	3.2	PT3A
Tilley	M	70	Adenocarcinoma	Prostate	3+4=7	14.0	PT3A
Tilley	M	64	Adenocarcinoma	Prostate	3+4=7	5.8	PT3A
Tilley	M	64	Adenocarcinoma	Prostate	3+4=7	5.7	PT3A
Tilley	M	60	Adenocarcinoma	Prostate	3+4=7	11.0	PT2C
Tilley	M	74	Adenocarcinoma	Prostate	3+4=7	28.0	PT3A
Tilley	M	68	Adenocarcinoma	Prostate	4+5=9	13.0	PT3A
Tilley	M	74	Adenocarcinoma	Prostate	3+4=7	9.0	PT2C
Tilley	M	59	Adenocarcinoma	Prostate	3+4=7	3.5	PT3C
Tilley	M	60	Adenocarcinoma	Prostate	3+4=7	9.0	PT3A
Tilley	M	65	Adenocarcinoma	Prostate	3+4=7	4.8	PT3A
Tilley	M	75	Adenocarcinoma	Prostate	4+3=7	10.0	PT3A
Tilley	M	71	Adenocarcinoma	Prostate	4+5=9	3.6	PT3B
Tilley	M	57	Adenocarcinoma	Prostate	3+4=7	5.8	PT3A
Tilley	M	68	Adenocarcinoma	Prostate	3+4=7	4.5	PT2C
Tilley	M	65	Adenocarcinoma	Prostate	3+4=7	6.9	PT3A
Tilley	M	53	Adenocarcinoma	Prostate	3+4=7	4.3	PT3A
Tilley	M	60	Adenocarcinoma	Prostate	3+4=7	8.0	PT3A
Tilley	M	67	Adenocarcinoma	Prostate	3+4=7	7.1	PT3A
Tilley	M	60	Adenocarcinoma	Prostate	3+4=7	5.0	PT2C
Tilley	M	57	Adenocarcinoma	Prostate	4+3=7	11.0	PT2C
Raj	M	64	Adenocarcinoma	Prostate	4+4=8	5.7	pT4
Raj	M	64	Adenocarcinoma	Prostate	4+3=7	8.3	pT2c
Raj	M	54	Adenocarcinoma	Prostate	3+4=7	6.9	pT2c
Raj	M	51	Adenocarcinoma	Prostate	4+5=9	11.0	pT3b
Raj	M	50	Adenocarcinoma	Prostate	5+5=10	16.0	pT3b
Raj	M	61	Adenocarcinoma	Prostate	4+3=7	12.0	pT3b
Raj	M	52	Adenocarcinoma	Prostate	4+5=9	32.0	pT3b
Raj	M	66	Adenocarcinoma	Prostate	4+5=9	3.0	pT3a
Raj	M	59	Adenocarcinoma	Prostate	3+4=7	4.6	pT2c
Raj	M	66	Adenocarcinoma	Prostate	4+3=7	7.4	pT2c
Knudsen	M	unknown	Adenocarcinoma	Prostate	4+3=7	4.3	
Knudsen	M	50	Adenocarcinoma	Prostate	3+3=6	5.7	
Knudsen	M	60	Adenocarcinoma	Prostate	3+4=7	6.4	
Knudsen	M	65	Adenocarcinoma	Prostate	3+4=7	4.5	

Laboratory	Gender	Age	Histological type	Site	Grade	UICC Stage	Receptor status
Tilley	F	66	infiltrating carcinoma, no special type	Breast	3	IB	ER+ PR+ HER2-
Tilley	F	72	infiltrating carcinoma, no special type	Breast	2	IIIA	ER+ PR+ HER2-
Tilley	F	81	infiltrating carcinoma, no special type	Breast	2	IA	ER+ PR+ HER2-
Tilley	F	54	invasive carcinoma of no special type	Breast	3	I	ER+ PR+ HER2+/-
Tilley	F	70	infiltrating carcinoma, no special type	Breast	3	IA	ER+ PR+ HER2-
Tilley	F	83	invasive carcinoma of no special type	Breast			ER+ PR+ HER2-
Tilley	F	56		Breast	1	IIB	
Tilley	F	63	invasive mucinous carcinoma	Breast	2	IIA	ER+ PR+ HER2-
Tilley	F	54	invasive carcinoma of no special type	Breast	2	I	ER+ PR+ HER2-
Tilley	F	56	infiltrating lobular carcinoma with mixed ductal differentiation	Breast	2	IIA	ER+ PR+ HER2-
Tilley	F	78	invasive lobular carcinoma	Breast	2	IIB	ER- PR- HER2+
Tilley	F	61	invasive carcinoma of no special type	Breast	3	I	ER+ PR+ HER2-
Tilley	F	51	Tubular carcinoma	Breast	1	I	ER+ PR- HER2-
Tilley	F	64	invasive carcinoma, mixed lobular and ductal type	Breast	2	I	ER+ PR+ HER2-

Supplementary Table 2. Summary of antibodies and conditions for immunohistochemistry.

Laboratory	Tissue	Antigen	Antigen Retrieval	Blocking Solution	Primary Antibody	Secondary Antibody
Raj	Prostate<br>Renal	HIF1α	Reveal (Biocare)	5% goat serum<br>30 min	H1alpha67<br>mouse monoclonal<br>(Novus Biologicals)<br>1:25<br>60 min	Ventana<br>30 min
Tilley	Prostate	AR	Decloaker; Citrate Buffer pH 6.0	5% goat serum<br>30 min	AR N-20<br>rabbit polyclonal<br>(Santa Cruz)<br>1:200<br>overnight at 4°C	Dako<br>30 min
Tilley	Prostate	PSA	none	5% goat serum<br>30 min	PSA<br>rabbit polyclonal<br>(Dako)<br>1:1000<br>overnight at 4°C	Dako<br>30 min
Tilley	Breast	ERα	Decloaker; Citrate Buffer pH 6.0	5% goat serum<br>30 min	ERα ID5<br>mouse monoclonal<br>(Dako)<br>1:300<br>overnight at 4°C	Dako<br>30 min
Tilley	Breast	PR	Decloaker; Citrate Buffer pH 6.0	5% goat serum<br>30 min	PR (A/B forms)<br>16SAN27<br>mouse monoclonal<br>(Novacastra)<br>1:1000<br>overnight at 4°C	Dako<br>30 min
Tilley	Prostate/ Breast	Ki67	Decloaker; Citrate Buffer pH 6.0	5% goat serum<br>30 min	Ki67 MIB-1<br>mouse monoclonal<br>(Dako)<br>1:200<br>overnight at 4°C	Dako<br>30 min

Supplementary Table 3. Primer sequences/Taqman Probe Assay IDs.

**Gene**	**Forward 5’-3’**	**Reverse 5’-3’**
KLK3 (PSA)	ACCAGAGGAGTTCTTGACCCCAAA	CCCCAGAATCACCCGAGCAG
FKBP5	AAAAGGCCAAGGAGCACAAC	TTGAGGAGGGGCCGAGTTC
KLK2	GGTGGCTGTGTACAGTCATGGAT	TGTCTTCAGGCTCAAACAGGTTG
NKX3.1	CTGGCAGAGACCGAGCCAGAAAG	AGCGCTTCTGCGGCTGCTTAG
TMPRSS2	GACCAAGAACAATGACATTGCG	GTTCTGGCTGCAGCATCATG
PPIA	GCATACGGGTCCTGGCAT	ACATGCTTGCCATCCAACC
L19	TGCCAGTGGAAAAATCAGCCA	CAAAGCAAATCTCGACACCTTG
TUBA1B	CCTTCGCCTCCTAATCCCTA	CCGTGTTCCAGGCAGTAGA
ALAS1	AGATCAAAGAAACCCCTCCG	AGCTGTGTGCCATCTGGACT
GAPDH	TGCACCACCAACTGCTTAGC	GGCATGGACTGTGGTCATGAG
**Gene**	**Taqman Probe Assay ID**	
PGR	Hs01556702_m	
IPO8	Hs00183533_m1	
PUM1	Hs00472881_m1	

Supplementary Table 4. Number of ER binding peaks identified by Mac Peak caller in explants (n=3) treated with E2 (estrodial) or E2 plus R5020 (progesterone receptor agonist).

Tumor ID	Factor	Treatment	Mac Peaks
1 T_206	ER	E2	56,723
2 T_206	ER	E2 + R5020	68,212
3 T_211	ER	E2	39,960
4 T_211	ER	E2 + R5020	25,713
5 T_213	ER	E2	8,613
6 T_213	ER	E2 + R5020	80,289

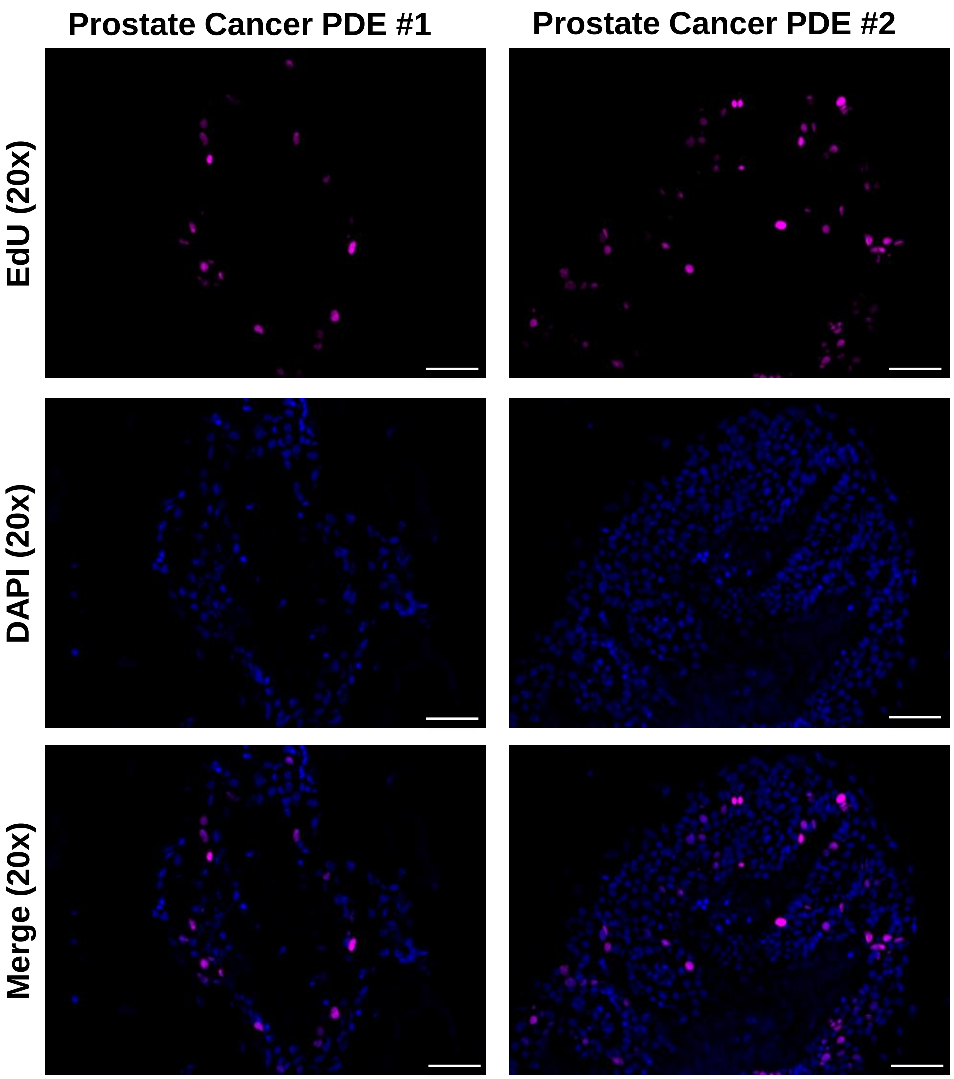

Supplementary Figure 1: Representative images of EdU staining in prostate cancer PDEs. After 24 h of culture, explant media was replaced with fresh media containing a final concentration of 10 µM EdU and cultured for another 24 h. Cellular uptake of EdU was stained by Click-iT EdU Imaging Kits (Thermo Fisher Scientific) following the manufacture's instruction. Magnification 20x, scale bars represent 50 µm.

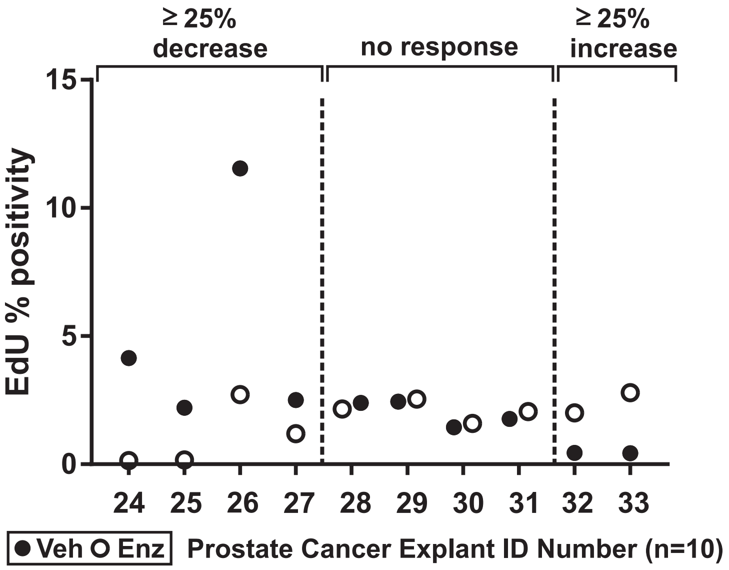

Supplementary Figure 2: Quantitation of EdU staining in prostate cancer PDEs (n=10) following 48h culture with vehicle control or enzalutamide (10μM). A response to enzalutamide was considered significant when treatment induced a change from vehicle of ≥25%.
